# Dextran sulfate sodium-induced colitis alters stress-associated behaviour and neuropeptide gene expression in the amygdala-hippocampus network of mice

**DOI:** 10.1038/srep09970

**Published:** 2015-06-12

**Authors:** Florian Reichmann, Ahmed Mostafa Hassan, Aitak Farzi, Piyush Jain, Rufina Schuligoi, Peter Holzer

**Affiliations:** 1 Research Unit of Translational Neurogastroenterology, Institute of Experimental and Clinical Pharmacology, Medical University of Graz, Graz, Austria

## Abstract

Psychological stress causes disease exacerbation and relapses in inflammatory bowel disease (IBD) patients. Since studies on stress processing during visceral inflammation are lacking, we investigated the effects of experimental colitis as well as psychological stress on neurochemical and neuroendocrine changes as well as behaviour in mice. Dextran sulfate sodium (DSS)-induced colitis and water avoidance stress (WAS) were used as mouse models of colitis and mild psychological stress, respectively. We measured WAS-associated behaviour, gene expression and proinflammatory cytokine levels within the amygdala, hippocampus and hypothalamus as well as plasma levels of cytokines and corticosterone in male C57BL/6N mice. Animals with DSS-induced colitis presented with prolonged immobility during the WAS session, which was associated with brain region-dependent alterations of neuropeptide Y (NPY), NPY receptor Y1, corticotropin-releasing hormone (CRH), CRH receptor 1, brain-derived neurotrophic factor and glucocorticoid receptor gene expression. Furthermore, the combination of DSS and WAS increased interleukin-6 and growth regulated oncogene-α levels in the brain. Altered gut-brain signalling in the course of DSS-induced colitis is thought to cause the observed distinct gene expression changes in the limbic system and the aberrant molecular and behavioural stress responses. These findings provide new insights into the effects of stress during IBD.

Inflammatory bowel disease (IBD) is a major health concern with a continuously rising incidence and prevalence^1^. Importantly, disease processes during IBD are not restricted to the intestinal tract and can affect other organs including the brain. In fact, IBD is associated with an increased risk of primary psychiatric diseases such as depression and anxiety disorders, aggravating the disease burden of the patients^2^. Furthermore, psychological stress reduces the quality of life and exacerbates the disease course of IBD patients^3^.

To model IBD in animals, dextran sulfate sodium (DSS)-induced colitis, a well-established rodent model of ulcerative colitis, is frequently used^4^. Studies utilizing this paradigm suggest a role of various stressful events in colitis development and exacerbation. For example, 4 days of restraint stress before DSS treatment shortens the latency to colitis development^5^, while chronic psychosocial stress increases colitis severity^6^. However, other reports suggest that certain forms of stress can be beneficial in the course of colitis. Acute water avoidance stress (WAS), a mild psychological stress paradigm^7^, decreases acetic acid colitis severity^8^ while repeated WAS alleviates DSS colitis-induced emotional-affective disturbances indicating a beneficial effect of stress exposure^9^.

Since stress is perceived and processed by the brain, it appears that stress-induced signals from the brain modulate gut function in health and disease. However, brain-gut communication is bidirectional, and recent work has emphasized a role of the gut-brain axis in the course of IBD^10^. Accordingly, the processing of intestinal signals in the brain is altered in patients with chronic gastrointestinal inflammation as deduced from altered neuronal activation patterns assessed by functional magnetic resonance imaging^11^. In a recent study we also found neuronal activation changes in the brain of mice with DSS-induced colitis, using c-Fos as a marker of neuronal activation. After a single session of WAS neuronal activation in the limbic system was blunted in mice with colitis^12^.

To analyse the mechanistic basis of these findings, the current study set out to explore stress-induced neuropeptide dynamics in the limbic system, which may be relevant to the interaction between internal inflammatory stress and external psychological stress in mice. We hypothesized that DSS colitis modifies the WAS-induced cerebral expression of stress-related neuropeptide systems. Therefore, we investigated the activity of the neuropeptide Y and corticotropin-releasing hormone systems, which have opposing roles during the stress response^13^. Specifically, corticotropin-releasing hormone (CRH) promotes activation of the hypothalamic-pituitary-adrenal (HPA) axis and induces stress-associated behavioural disturbances^14^, while neuropeptide Y (NPY) promotes stress resilience, the ability to cope with stress^15^. These actions of the neuropeptides also depend on the receptor activated. Thus, corticotropin-releasing hormone receptor 1 (CRHR1) appears to be responsible for the initiation of the stress response, while corticotropin-releasing hormone receptor 2 (CRHR2) activation is involved in the recovery phase after stress exposure^16^. In contrast, neuropeptide Y receptor Y1 (NPY1R) stimulation promotes stress resilience, while neuropeptide Y receptor Y2 (NPY2R) activation reduces NPY release into the synaptic cleft^15^,^17^. Brain-derived neurotrophic factor (BDNF) expression was measured as a marker of stress-induced neuronal plasticity^18^ and glucocorticoid receptor (NR3C1) expression was analysed as an indicator of stress hormone action within the brain^19^. To evaluate the effects of colitis on the stress response at the functional level, we assessed whether DSS treatment alters behaviour during WAS. Proinflammatory cytokine levels in plasma and brain as well as circulating corticosterone levels were measured as potential mediators of the central effects induced by DSS and/or WAS.

## Materials and Methods

### Ethical statement

All animal procedures were approved by an ethical committee at the Federal Ministry of Science and Research of the Republic of Austria (BMWF-66.010/0119-II/3b/2011 and BMWF-66.010/0037-II/3b/2013) and conducted according to the Directive of the European Parliament and of the Council of 22 September 2010 (2010/63/EU).

### Study design

**Figure 1:**
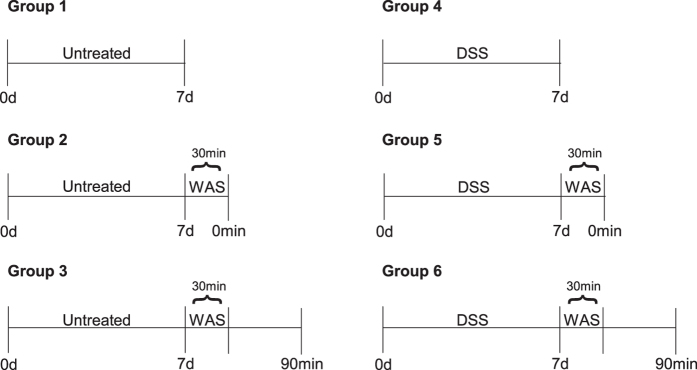
Experimental groups and timeline. Mice from group 1–3 received plain drinking water (untreated), while animals from group 4-6 were treated with dextran sulfate sodium (DSS; 2%, added to the drinking water) for 7 days. After the 7-day treatment period, animals were either sacrificed for organ collections (groups 1 and 4) or exposed to water avoidance stress (WAS; groups 2,3,5 and 6) for 30 min. The WAS-exposed mice were sacrificed either immediately (0 min; groups 2 and 5) or 90 min after the end of the 30-min WAS session (groups 3 and 6).

30 male 10-week old C57BL/6N mice, obtained from Charles River (Sulzfeld, Germany), were treated for 7 days with DSS (molecular weight 36-50 kDa; MP Biomedicals, Illkirch, France; 2%, added to the drinking water) to induce colitis, while another 30 untreated animals received plain drinking water^12^. After this treatment period, 20 mice from each group were submitted to a single 30-min session of WAS, while 10 mice from each group remained unstressed. With this design, a total of 6 experimental groups (n = 10/group) were used (Fig. 1):

1. Untreated mice sacrificed without WAS exposure
2. Untreated mice sacrificed immediately after WAS
3. Untreated mice sacrificed 90 min after WAS
4. DSS-treated mice sacrificed without WAS exposure
5. DSS-treated mice sacrificed immediately after WAS
6. DSS-treated mice sacrificed 90 min after WAS

Before the start of any experiment, mice were allowed to habituate to the in-house animal facility under controlled conditions of temperature (set point 21 °C) and air humidity (set point 50%) and under a 12 h light/dark cycle (lights on at 0600 h and lights off at 1800 h) for 2 weeks. To minimize any influence of circadian rhythm on our chosen readouts, experiments were performed exclusively during the first half of the light phase (0800 h to 1200 h) and the order of testing was designed in a way, which ensured that different treatment groups were tested during comparable circadian phases. Stress-associated behaviour during the WAS session was recorded in the mice sacrificed immediately after WAS. Molecular readouts in brain, blood and colon were taken from all experimental groups.

### Evaluation of experimental colitis

At the end of the 7-day treatment period a disease activity score (DAS) was calculated to assess the physical status of the animals^20^. The score included 3 parameters: weight difference between start and end of dextran sulfate sodium treatment (score 0: weight increase ≥ 1 g, score 1: weight increase < 1 g, score 2: weight decrease < 1 g, score 3: weight decrease ≥ 1 g), stool consistency (score 0: normal stool, score 1: soft but formed stool, score 2: loose stool) and presence of blood in the perianal region (score 0: no traces of blood in the perianal region, score 1: traces of blood in the perianal region, score 2: bloody perianal region). Accordingly, the minimum score was 0 and the maximum score was 7. In addition, colon length was recorded as a further index of colitis^20^.

### Water avoidance stress

As previously described^12^, mice were placed on a small plastic block (6 × 3 x 6 cm, length x width x height) in the centre of a water-filled tank (61×40 x 22 cm, length x width x height) for 30 min, the level of the water (25 °C) in the tank being just beneath the block. Immediately after submission to WAS the behaviour of mice was recorded for 10 min to evaluate their behavioural reaction to stress and analysed by a blinded investigator. Time spent moving on the platform (activity), time spent immobile and time spent grooming was semi-automatically quantified with the VideoMot 2 software (TSE systems, Bad Homburg, Germany).

### Blood collection

For blood sampling mice were deeply anesthetized with an overdose of pentobarbital (150 mg/kg injected intraperitoneally). Blood was collected by cardiac puncture between 09:00 and 12:00 with syringes containing sodium citrate (3.8%; w/v) as an anticoagulant. Following centrifugation for 10 min at 4 °C and 7000 rpm, blood plasma for corticosterone and cytokine measurements was collected and stored at -20 °C and -70 °C until assay, respectively.

### Circulating corticosterone

As previously described^12^, plasma corticosterone was determined with an enzyme immunoassay kit (Assay Designs, Ann Arbor, Michigan, USA). According to the manufacturer’s specifications, the sensitivity of the assay is 27 pg/ml, and the intra- and inter-assay coefficient of variation amounts to 7.7 and 9.7%, respectively.

### Cytokines

Concentrations of interleukin (IL)-1β, IL-6, IL-17A, IL-18, tumour necrosis factor-α (TNF-α) and growth regulated oncogene-α (GRO-α) were quantified in duplicates in plasma and brain tissue using ProcartaPlex™ Multiplex immunoassays (eBioscience, San Diego, CA, USA). In order to use this assay for the measurement of cytokines in the brain, the assay was validated by the company. No crossreactivity between the selected cytokines was detected. The inter- and intraassay coefficient of variation amounted to 7-10% and 7-15%, respectively. The cerebral levels of cytokines were measured in the hippocampus, amygdala and hypothalamus. These microdissected brain areas were homogenized using the Procarta Cell Lysis buffer (eBioscience). The protein concentration in each sample was measured with the Pierce BCA Protein Assay Kit (Thermo Scientific, Waltham, MA, USA). Brain samples were diluted with sample dilution buffer provided by the kit to yield a concentration of 10 mg protein/ml, 7.5 mg protein/ml and 6 mg protein/ml for hippocampus, amygdala and hypothalamus, respectively. According to the manufacturer’s instructions cytokine concentrations were determined using analyte-specific magnetic beads coated with target-specific antibodies. Target-specific fluorescent signals were measured with the Bio-Plex 200 multiplex suspension array system employing Luminex xMAP-technology in combination with the Bio-Plex 5.0 Software (Bio-Rad, Hercules, CA, USA). Standard curves for each analyte were generated by using the reference analyte concentration supplied, and concentrations were calculated with a five-parameter logistic curve-fitting method. Values below the detection limit were set as zero. According to the manufacturer, the assay sensitivity for the respective cytokines is: IL-1β - 0.14 pg/ml, IL-6 - 0.21 pg/ml, TNF-α - 0.39 pg/ml, IL-17A - 0.08 pg/ml, IL-18 - 9.95 pg/ml and GRO-α - 0.05 pg/ml.

### Mouse brain microdissection

As previously described^21^, freshly excised mouse brains were frozen in -70 °C cold 2-methyl butane (Carl Roth, Karlsruhe, Germany) and cut manually into approximately 1mm thin coronal slices from which brain areas of interest were isolated. For this purpose the sliced brain was placed on a cold plate (Weinkauf Medizintechnik, Forchheim, Germany) set at -20 °C, on which hippocampus, amygdala and hypothalamus were microdissected under a stereomicroscope using precooled iris spatulae (Fine Science Tools, Heidelberg, Germany). Hippocampal tissue was collected across the whole rostrocaudal extent starting from the limit of the hippocampal formation (Bregma: –0.94) to the caudal end of the dentate gyrus (Bregma: –4.04). The hippocampal tissue from several slices was pooled and thus contained the ventral and dorsal subparts as well as the dentate gyrus and the CA regions. The microdissection of the amygdala was performed across the whole rostrocaudal extent of the amygdalar complex (Bregma: -0.58 to Bregma: -2.54). The amygdalar tissue taken contained all major amygdalar subnuclei (medial, central, basolateral, basomedial, lateral and cortical). The hypothalamic tissue collected ranged from the beginning of the preoptic area (Bregma: + 0.26) to the end of the mammillary bodies (Bregma: -2.92). Isolated brain areas were collected in homogenization tubes kept on dry ice and stored at -70 °C until further processing.

### Real-time reverse transcription PCR

Brain tissues used for real-time reverse transcription PCR were homogenized with a Precellys 24 homogenizer (Peqlab Biotechnology, Polling, Austria) and RNA was extracted with the RNeasy Lipid Tissue Mini kit (Qiagen, Vienna, Austria). The RNA extraction method was tested for quality on the BioAnalyzer BA2100 (Agilent, Foster City, CA, USA) with the RNA 6000 Nano LabChip Kit (catalogue number 5067-1511, Agilent). The RIN (RNA Integrity Number) of all tested samples ranged between 8.3 and 9.9. After extraction, the RNA concentration was measured and 1 μg RNA/sample submitted to a reverse transcription reaction using the high capacity cDNA reverse transcription kit (Applied Biosystems, Foster City, CA, USA). For relative quantification of mRNA levels within a given brain area, specific primers for *Npy*^22^, *Npy1r* (Harvard primer bank^23^; ID: 6754882a1), *Npy2r* (Harvard primer bank, ID: 148277108c2), *Crh* (designed with Primer-BLAST), *Bdnf* (designed with Primer-BLAST), *Nr3c1* (Harvard primer bank, ID: 121247452c1) and peptidylprolyl isomerase A (*Ppia*) were used at a concentration of 1 μM each (Table 1) together with the SsoAdvanced Universal SYBR Green Supermix (Bio-Rad, Hercules, CA, USA). DNA sequencing and the Basic Local Alignment Search Tool of the National Center of Biotechnology Information^24^ confirmed the amplification of the correct base pair sequence. Primers for *Crhr1 (*Assay ID: qMmuCID0010106) and *Crhr2 (*Assay ID: qMmuCID0015787) were purchased from Bio-Rad. Samples were measured in triplicates and *Ppia* was used as the reference gene. The PCR run was performed on a Bio-Rad CFX Connect Real-Time PCR Detection System (Bio-Rad) and the respective CFX Manager software 3.1(Bio-Rad). Group differences were expressed as fold changes according to the 2^-ΔΔCt^ method using the mean value of the untreated/no WAS group as the calibrator^25^.

### Colonic myeloperoxidase content

The tissue levels of myeloperoxidase were used to quantify inflammation-associated infiltration of neutrophils and monocytes into the tissue. As previously described^12^, full-thickness pieces of the distal colon were excised, shock-frozen in liquid nitrogen and homogenized. Samples were measured with an enzyme-linked immunosorbent assay kit specific for the rat and mouse protein (Hycult Biotechnology, Uden, The Netherlands). The sensitivity of this assay is 1 ng/ml at an intra- and inter-assay variation of around 10%.

### Statistics

Statistical data analysis was performed on SPSS 22.0 (SPSS Inc., Chicago, IL, USA) and SigmaPlot 12.5 (Systat Software Inc, San Jose, CA, USA). For all analyses the normal distribution of the data was assessed with the Shapiro-Wilk test, while the homogeneity of variances was analysed with the Levene test. Data not normally distributed or with unequal variances were log-transformed (MPO, corticosterone, hippocampal and hypothalamic *Npy*, amygdalar and hypothalamic *Npy1r*, hippocampal and hypothalamic *Npy2r*, amygdalar *Crh*, amygdalar *Crhr2*, hypothalamic *Bdnf*, hypothalamic *Nr3c1*) or rank-transformed (DAS) to meet ANOVA assumptions. Data consisting of one variable and 2 factors (i.e. stress time point and colitis) were analysed by two-way ANOVA. In case of a significant interaction of the 2 factors or for main effect analysis, post-hoc tests were performed (Tukey HSD). Cytokine data, which could not be successfully log or rank-transformed to meet ANOVA assumptions, were analysed using the Kruskal-Wallis test followed by the Mann-Whitney-U test for pairwise comparisons followed by Bonferroni correction. Data consisting of one variable but only two groups were analysed with Student’s t test. Probability values of p < 0.05 were regarded as statistically significant.

## Results

### Dextran sulfate sodium treatment induces colonic inflammation and alters hypothalamic-pituitary-adrenal axis activity

**Figure 2:**
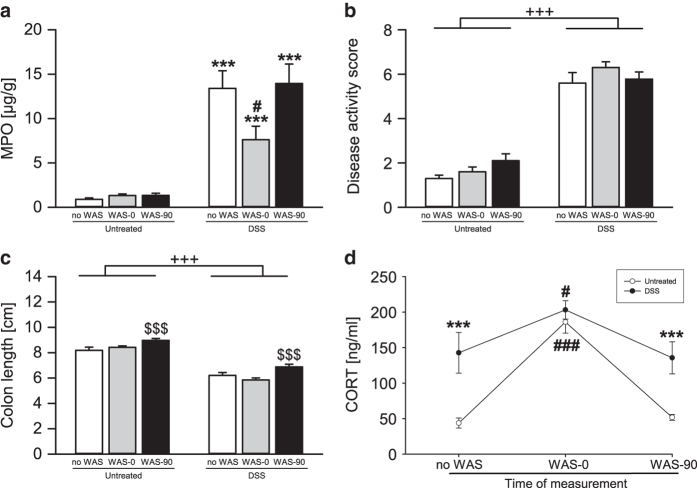
Dextran sulfate sodium (DSS) treatment induces colonic inflammation and increases circulating corticosterone (CORT) levels. Mice treated with DSS for 7 days had increased (**a**) colonic myeloperoxidase (MPO) levels, (**b**) a higher disease activity score and (**c**) decreased colon length. Water avoidance stress (WAS) had a short-lasting effect on colonic MPO content, increased colon length, but did not alter the disease activity score. (**d**) Basal (no WAS) and post-stress (WAS-90) CORT levels were elevated by DSS treatment, while WAS increased CORT levels immediately (WAS-0), but not 90 min, post-WAS. The values represent means + SEM or means±SEM, n = 7-10. ***P < 0.001 versus same stress exposure of Untreated; ^#^P < 0.05, ^###^P < 0.001 versus no WAS of the same treatment;^+++^P < 0.001 for main effect: DSS versus Untreated independently of stress exposure; ^$$$^P < 0.001 for main effect: WAS-90 versus no WAS independently of DSS treatment.

DSS treatment (F_(1,51)_ = 176.851; P < 0.001) but not WAS elevated colonic MPO levels with a significant interaction between these factors (F_(2,51)_ = 4.119; P = 0.022). Post hoc analysis revealed a transient anti-inflammatory effect of WAS as it decreased the colonic MPO content of DSS-treated mice immediately (0 min) but not 90 min after WAS exposure (Fig. 2a). DSS treatment also increased the DAS (F_(1,54)_ = 193.728; P < 0.001) and shortened colon length (F_(1,53)_ = 197.686; P < 0.001), while WAS (F_(2,53)_ = 10.129; P < 0.001) increased colon length 90 min, but not immediately, after WAS exposure (Fig. 2b,c). Furthermore, HPA axis activity was altered by DSS treatment (F_(1,54)_ = 39.186; P < 0.001) and WAS (F_(2,54)_ = 31.263; P < 0.001) with a significant interaction between the 2 factors (F_(2,54)_ = 7.753; P = 0.001). Specifically, DSS-treated mice had higher basal as well as post-stress corticosterone levels (90 min after WAS) than untreated animals (Fig. 2d). WAS led to a transient corticosterone rise in both treatment groups, with higher levels present immediately but not 90 min after stress exposure.

### Colitis modifies stress-related behaviour

**Figure 3:**
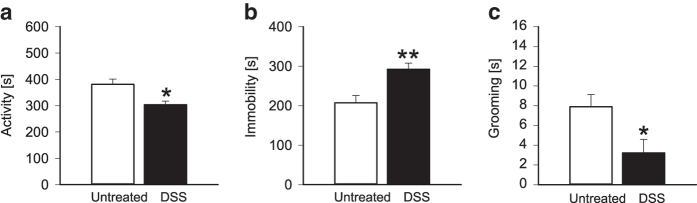
Colonic inflammation alters behaviour of mice. During water avoidance stress exposure, mice treated with dextran sulfate sodium (DSS) for 7 days showed (**a**) less activity, (**b**) more immobility and (**c**) less grooming behaviour than untreated animals (Untreated). The values represent means + SEM, n = 7-9. *P < 0.05, **P < 0.01 versus Untreated.

Analysis of the behaviour during WAS revealed that mice with colitis were less willing to explore (Fig. 3a), spent more time being immobile (Fig. 3b) and were less occupied with grooming behaviour (Fig. 3c) than untreated animals.

### Colitis increases circulating proinflammatory cytokine levels

DSS treatment increased circulating IL-6, IL-18, TNF-α and GRO-α levels in both unstressed mice and mice exposed to WAS (Table 2). Furthermore, combined DSS + WAS treatment elevated IL-17A levels compared to untreated WAS-exposed mice. WAS failed to enhance cytokine levels in untreated animals and circulating IL-1β levels were below the detection limit.

### The combination of colitis and water avoidance stress increases cerebral IL-6 and GRO-α levels

Cerebral IL-6 levels were increased in WAS-exposed animals with colitis in all brain regions examined compared to untreated WAS-exposed animals (Table 2). Furthermore, GRO-α levels were enhanced in the hypothalamus and hippocampus of WAS-exposed animals with colitis, whereas in unstressed mice with colitis GRO-α levels were elevated only in the hypothalamus. The concentrations of IL-1β, IL-17A, IL-18 and TNF-α were similar in all treatment groups.

### Colitis and water avoidance stress modify NPY and CRH-related gene expression in the hypothalamus and limbic system in a region-dependent manner

#### Hypothalamus

**Figure 4:**
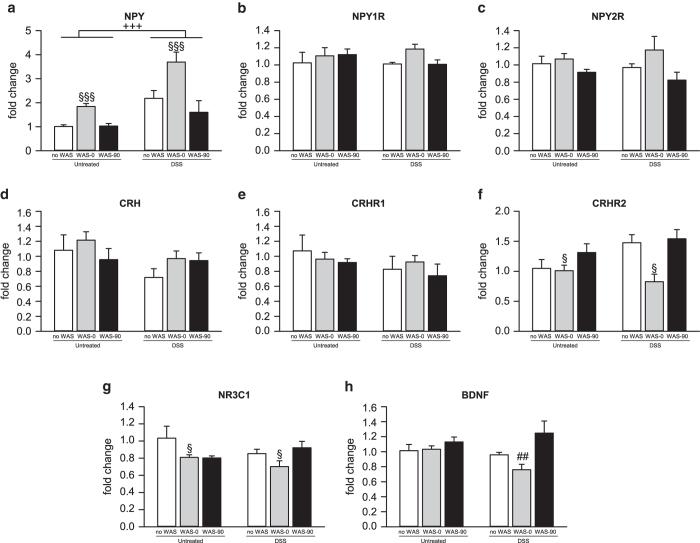
Colonic inflammation and acute psychological stress lead to distinct changes in hypothalamic gene expression. The graph displays the effects of a 7-day dextran sulfate sodium (DSS) treatment and water avoidance stress (WAS) in mice on mRNA levels for (**a**) neuropeptide Y (NPY), (**b**) NPY receptor Y1 (NPY1R), (**c**) NPY receptor Y2 (NPY2R), (**d**) corticotropin-releasing hormone (CRH), (**e**) CRH receptor 1 (CRHR1), (**f**) CRH receptor 2 (CRHR2), (**g**) glucocorticoid receptor (NR3C1) and (**h**) brain-derived neurotrophic factor (BDNF). Parameters were measured in unstressed mice (no WAS) as well as immediately (WAS-0) and 90 min (WAS-90) after exposure to a 30-min WAS session following the 7-day treatment. The values are expressed as fold changes normalized to Untreated/no WAS and represent means + SEM, n = 5-7. ^##^P < 0.01 versus same stress exposure of Untreated;^+++^P < 0.001 for main effect: DSS versus Untreated independently of stress exposure; ^§^P < 0.05, ^§§§^P < 0.001 for main effect: WAS-0 versus no WAS independently of DSS treatment.

DSS treatment increased hypothalamic *Npy* expression (F_(1,27)_ = 22.575; P < 0.001) which, in addition, was elevated by WAS (F_(2,27)_ = 16.309; P < 0.001) immediately, but not 90 min, after stress exposure (Fig. 4a). In contrast, both factors failed to alter the transcription rate of the corresponding *Npy1r* and *Npy2r* (Fig. 4b,c). WAS, but not colitis, also altered the activity of the hypothalamic CRH system (Fig. 4d–f). This was reflected by a decrease of *Crhr2* mRNA levels immediately, but not 90 min, after the WAS session (F_(2,28)_ = 8.455; P = 0.001; Fig. 4f). Hypothalamic *Nr3c1* mRNA levels were decreased by WAS immediately, but not 90 min after stress exposure (F_(2,28)_ = 4.257; P = 0.024; Fig. 4g). *Bdnf* expression was influenced by WAS (F_(2,28)_ = 7.402; P = 0.003), but not colitis, with a significant interaction between these factors (F_(2,28)_ = 3.888; P = 0.032). Post-hoc analysis revealed that the combination of DSS treatment with WAS exposure reduced hypothalamic *Bdnf* expression immediately after WAS compared to WAS-exposed untreated mice (Fig. 4h).

#### Amygdala

**Figure 5:**
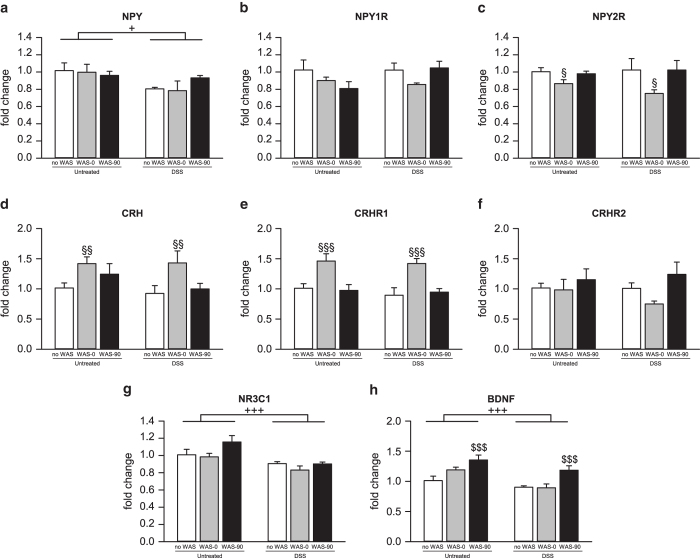
Colonic inflammation and acute psychological stress lead to distinct changes in amygdalar gene expression. The graph displays the effects of dextran sulfate sodium (DSS) treatment and water avoidance stress (WAS) in mice on mRNA levels for (**a**) neuropeptide Y (NPY), (**b**) NPY receptor Y1 (NPY1R), (**c**) NPY receptor Y2 (NPY2R), (**d**) corticotropin-releasing hormone (CRH), (**e**) CRH receptor 1 (CRHR1), (**f**) CRH receptor 2 (CRHR2), (**g**) glucocorticoid receptor (NR3C1) and (**h**) brain-derived neurotrophic factor (BDNF). Parameters were measured in unstressed mice (no WAS) as well as immediately (WAS-0) and 90 min (WAS-90) after exposure to a 30-min WAS session following the 7-day DSS treatment. The values are expressed as fold changes normalized to Untreated/no WAS and represent means + SEM, n = 5-7. + P < 0.05,^+++^P < 0.001 for main effect: DSS versus Untreated independently of stress exposure; ^§^P < 0.05, ^§§^P < 0.01, ^§§§^P < 0.001 for main effect: WAS-0 vs. no WAS independently of DSS treatment; ^$$$^, P < 0.001 for main effect: WAS-90 versus no WAS independently of DSS treatment.

*Npy* expression in the amygdala was decreased by DSS treatment (F_(1,28)_ = 4.778; P = 0.037; Fig. 5a). In contrast, *Npy1r* expression was not affected by DSS treatment and WAS exposure (Fig. 5b), while *Npy2r* expression was reduced by WAS (F_(2,28)_ = 5.613; P = 0.009) as seen immediately, but not 90 min, post-stress exposure (Fig. 5c). Furthermore, WAS enhanced the expression of amygdalar *Crh* (F_(2,28)_ = 6.129; P = 0.006) and *Crhr1* (F_(2,28)_ = 17.237; P < 0.001) as observed immediately, but not 90 min, after the WAS session (Fig. 5d,e), but did not influence *Crhr2* expression (Fig. 5f). In contrast, both amygdalar *Nr3c1* (F_(1,28)_ = 17.878; P < 0.001) and *Bdnf* expression (F_(1,28)_ = 13.798; P < 0.001) were attenuated by colitis (Fig. 5g,h) and *Bdnf* expression was increased by WAS (F_(2,28)_ = 11.956; P < 0.001) 90 min, but not immediately after stress exposure.

#### Hippocampus

**Figure 6:**
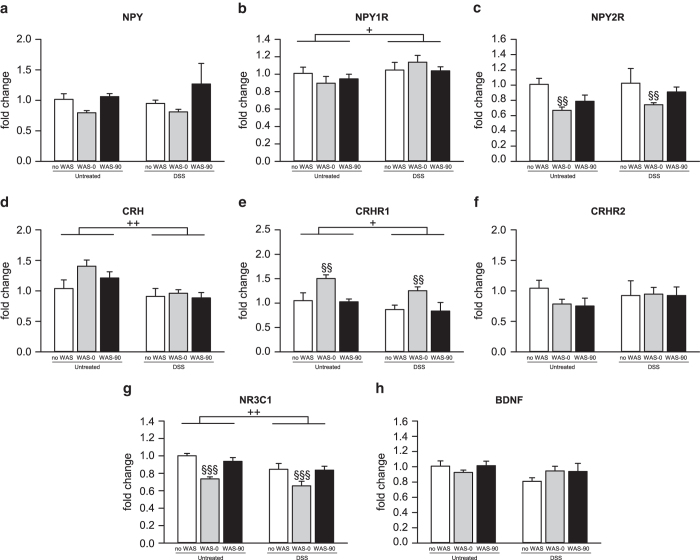
Colonic inflammation and acute psychological stress lead to distinct changes in hippocampal gene expression. The graph displays the effects of dextran sulfate sodium (DSS) treatment and water avoidance stress (WAS) in mice on mRNA levels for (**a**) neuropeptide Y (NPY), (**b**) NPY receptor Y1 (NPY1R), (**c**) NPY receptor Y2 (NPY2R), (**d**) corticotropin-releasing hormone (CRH), (**e**) CRH receptor 1 (CRHR1), (**f**) CRH receptor 2 (CRHR2), (**g**) glucocorticoid receptor (NR3C1) and (**h**) brain-derived neurotrophic factor (BDNF). Parameters were measured in unstressed mice (no WAS) as well as immediately (WAS-0) and 90 min (WAS-90) after exposure to a 30-min WAS session following the 7-day DSS treatment. The values are expressed as fold changes normalized to Untreated/no WAS and represent means + SEM, n = 5–7. + P < 0.05,^++^P < 0.01 for main effect: DSS versus Untreated independently of stress exposure; ^§§^P < 0.01, ^§§§^P < 0.001 for main effect: WAS-0 versus no WAS independently of DSS treatment.

Hippocampal *Npy* expression was neither influenced by WAS nor DSS treatment (Fig. 6a). However, *Npy1r* expression was increased by DSS treatment (F_(1,27)_ = 4.258; P = 0.049; Fig. 6b), while *Npy2r* mRNA levels were diminished by WAS (F_(2,27)_ = 6.242; P = 0.006) immediately, but not 90 min, after stress exposure (Fig. 6c). Hippocampal *Crh* (F_(1,27)_ = 12.096; P = 0.002) and *Crhr1* (F_(1,27)_ = 5.212; P = 0.031), but not *Crhr2*, expression was attenuated by DSS treatment (Fig. 6d–f). In contrast, *Crhr1* expression was elevated by WAS (F_(2,27)_ = 11.074; P < 0.001) immediately, but not 90 min, after WAS exposure (Fig. 5e), and hippocampal *Nr3c1* expression was blunted by colitis (F_(1,27)_ = 9.356; P = 0.005) and WAS (F_(2,27)_ = 16.235; P < 0.001) immediately, but not 90 min, after stress (Fig. 6g). The hippocampal *Bdnf* levels remained unaltered by WAS and DSS treatment (Fig. 6h).

## Discussion

Stress is a crucial factor in the course of chronic visceral inflammation, which points to a close pathogenic interaction between the brain and gut. However, studies on central adaptation processes contributing to disturbances of stress responsiveness, brain function and emotional-affective behaviour during visceral inflammation are largely lacking. Here we report that colitis alters the behavioural reaction to stress, a process accompanied by distinct region-dependent gene expression changes within the limbic system. Furthermore, we demonstrate that the combination of peripheral inflammation and water avoidance stress increases cerebral IL-6 and GRO-α content. Colitis also modifies HPA axis activity and increases several proinflammatory circulating cytokines.

The severity of DSS-induced colitis as judged by the increased colonic MPO content, increased DAS and shortened colon length is in line with previous reports^12^,^20^,^26^ and was rated as mild because the treatment paradigm used here does not cause substantial deterioration of the mucosal architecture^20^. Although chronic stress exacerbates DSS-induced colitis^6^, the colonic MPO content of DSS-treated mice was diminished immediately after acute WAS exposure. A similar anti-inflammatory effect of acute WAS has been found in the acetic acid colitis model^8^, which may be due to the anti-inflammatory action of glucocorticoids released by stress exposure. The WAS-induced increase of colon length, which occurs independently of DSS treatment, may arise from stress-induced sympathetic nerve activity which lowers smooth muscle tone of the colon and thus causes colonic relaxation^27^.

We have previously shown that WAS-induced neuronal activation of various brain regions, as measured by the expression of the immediate early gene c-Fos, largely differs between mice with and without colitis 90 min after stress exposure^12^. Here we were able to show that the behaviour during stress exposure was significantly changed in mice with colitis as reflected by decreased activity, increased immobility and attenuated grooming. These behavioural alterations may reflect impaired stress coping and resemble a depression-like phenotype, given that prolonged immobility is the major depression readout in the forced swim and tail suspension tests^28^,^29^. Reduced grooming reflects decreased self-care and motivation, another trait of depression-like behaviour^30^. Disturbances of depression-like behaviour associated with DSS-induced colitis have also been reported with the forced swim test^26^. However, this interpretation is not unequivocal because the immobility of DSS-treated mice during WAS exposure could also reflect enhanced anxiety, given that anxious mice tend to freeze during aversive situations. Increased anxiety of DSS-treated mice has in fact been demonstrated in previous reports^9^,^26^.

The colitis-induced molecular and behavioural changes are thought to result from altered gut-brain communication in which proinflammatory cytokines play a major role. As described previously, DSS-induced colitis enhanced the circulating levels of IL-6, TNF-α and GRO-α^31^. Circulating IL-17A levels increased only in mice with colitis exposed to WAS, which indicates that stress can promote cytokine formation. The presence of cytokines under conditions of colitis and stress was not confined to the periphery but also extended to the brain as shown for the first time in the present study. Thus, the combination of DSS treatment and WAS exposure enhanced the levels of IL-6 in the amygdala, hippocampus and hypothalamus and the levels of GRO-α in hippocampus and hypothalamus, whereas following DSS treatment only the GRO-α concentration in the hypothalamus was elevated. However, from this data it is not possible to decide whether the combination of stress and colitis enhances the passage of cytokines across the blood-brain barrier and/or triggers additional cytokine production within the brain^32^,^33^. It should be noted, however, that the treatment-related patterns of cytokine concentrations in the brain do not match with the patterns of plasma cytokine concentrations and other markers of colitis (MPO, DAS), suggesting a central effect of WAS to promote neuroinflammation. The increase in the cerebral IL-6 concentration may have a bearing on the concomitant rise of corticosterone levels as IL-6 can activate the HPA axis^34^ while GRO-α, a chemokine, promotes neuroinflammation by influencing microglial migration^35^.

The colitis-induced alteration of the behavioural reaction to stress was paralleled by changes in the neuroendocrine stress response as assessed by circulating corticosterone, an index of HPA axis activity^19^. DSS-induced colitis elevated corticosterone levels, a finding that was also reported in the trinitrobenzene sulfonic acid colitis model^36^. Given the anti-inflammatory properties of glucocorticoids, the enhanced plasma levels of corticosterone may be seen as an attempt to counteract inflammation and restore homeostasis. Since the peak corticosterone levels (measured immediately after the stress session) were similar in both treatment groups it would seem, however, that HPA axis reactivity to psychological stress remained intact during colitis. This conclusion is corroborated by the colitis-independent cessation of the stress-induced rise of circulating corticosterone 90 min after stress exposure.

Of the many neuropeptides implicated in stress processing, NPY and CRH take a special place as they are regulated by stress exposure, but have opposing effects on stress-related behaviour. While CRH induces anxiety and depression-like behaviour, NPY has anxiolytic and anti-depressant properties^37^. In the current study, colitis had many region-specific effects on these neuropeptide systems. Specifically, DSS-induced colitis increased hypothalamic *Npy* expression, which is in line with a colitis-induced enhancement of NPY release from the hypothalamic paraventricular nucleus^38^. In view of the colitis-induced weight loss^38^ and the crucial role of hypothalamic NPY in stimulating ingestion we hypothesize that *Npy* upregulation in the hypothalamus reflects a counterregulatory mechanism to restore energy homeostasis^39^.

In contrast, colitis blunted the expression of *Npy* in the amygdalar complex. In view of the essential role of the basolateral amygdala in fear and stress processing^15^,^40^, we hypothesize that the lower *Npy* expression within the amygdalar complex may explain why mice with colitis displayed a deficit in stress coping and affective behaviour during the WAS session. In the hippocampus, colitis did not alter *Npy* expression, but increased *Npy1r* and decreased *Crhr1* mRNA levels. These molecular changes did not translate into behavioural alterations, given that overexpression of hippocampal *Npy1r* has been reported to elicit modest anxiolysis and CRHR1 antagonism has anxiolytic effects^14^,^41^. It rather seems that the downregulation of *Npy* in the amygdalar complex, which is a key brain region for mitigating emotional-affective behaviour^42^, overrides molecular adaptations in other brain regions.

Colitis also decreased hippocampal *Crh* expression, which may reflect a negative feedback mechanism elicited by the elevated corticosterone concentrations in colitis. The reason for this region-dependent effect may be the particular high density of NR3C1s within the hippocampus^43^. A trend towards a reduction of *Crh* mRNA levels was also seen in the hypothalamus, which is in line with a previous report^36^. Hippocampal and amygdalar *Nr3c1* expression itself was also decreased by DSS treatment, an effect that may likewise be related to the elevated corticosterone concentrations in colitis. Since elevated corticosterone levels in the amygdala, through stimulation of NR3C1, induce anxiety in rats^44^, the behavioural alterations in DSS-treated mice may, at least in part, be due to a change in amygdalar glucocorticoid signalling. Colitis had also a region-dependent effect on amygdalar and hypothalamic *Bdnf* expression. As BDNF is a key modulator of neuronal plasticity^18^, impaired neuronal plasticity may potentially contribute to the behavioural alterations associated with colitis.

The effects of WAS on cerebral gene expression were remarkably similar among mice with and without colitis. *Npy* expression was selectively increased by WAS in the hypothalamus, an effect absent in the amygdala and hippocampus. This stress-induced increase of hypothalamic *Npy* expression has also been shown by others^22^,^45^. The hypothalamic *Npy* response was found to be rapid and short-lasting, which fits with the proposed role of NPY to counteract the biological effects of CRH during the stress response^40^. The finding that WAS did not significantly enhance hypothalamic *Crh* expression itself cannot be explained conclusively, unless the release of CRH in the hypothalamus is recorded and the dynamics of *Crh* expression are specifically monitored in the hypothalamic paraventricular nucleus which is the major source of cerebral CRH^46^. However, the observed hypothalamic *Crhr2* downregulation suggests a modulatory capacity of WAS on the CRH system in this area.

In the amygdala, however, WAS evoked a rapid and short-lasting increase of *Crh* mRNA levels. Such rapid *Crh* transcription changes after stress exposure have already been shown earlier in the hypothalamus. Specifically, Imaki *et al.*^47^ demonstrated an increase of Crh hnRNA in the hypothalamic paraventricular nucleus as early as 5 min after stress onset. The observed increase in amygdalar *Crh* expression may be a molecular correlate of stress-induced anxiety. This notion is supported by the anxiogenic effect of *Crh* overexpression in the rat amygdala^48^. The stress-induced alterations of *Npy* and *Crh* transcription were paralleled by distinct changes in receptor expression. While *Npy1r* was not affected by WAS, *Npy2r* expression decreased in the amygdala and hippocampus. As the NPY2R is an autoreceptor located at the presynaptic membrane and its activation reduces NPY synthesis and release^17^, *Npy2r* downregulation enhances NPY1R stimulation and thus facilitates stress coping^15^. In this argument, however, the increase of *Crhr1* expression in the amygdala and hippocampus needs to be considered because CRHR1 stimulation opposes the effect of NPY1R activation and induces anxiety^49^.

In addition, WAS decreased *Nr3c1* expression in the hypothalamus and hippocampus immediately after stress exposure, an effect related to enhanced NR3C1 stimulation through high corticosterone levels at this time point. This is consistent with the important roles which glucocorticoids play in stress responses at the level of the hippocampus and amygdala^19^,^42^. The elevated expression of *Bdnf* in the amygdala suggests that WAS promotes neuronal plasticity, which is in keeping with stress-induced synaptic remodelling in this brain area^50^.

In conclusion, the current study discloses new aspects of gut-brain and brain-gut signalling in the interaction of experimental colitis and psychological stress. Mice with colitis showed altered stress-associated behaviour, which is associated with colitis-induced alterations of *Npy*, *Npy1r*, *Crh*, *Crhr1*, *Bdnf* and *Nr3c1* gene expression in distinct regions of the brain. Elevated plasma levels of corticosterone and proinflammatory cytokines as well as increased concentrations of IL-6 and GRO-α in the brain are likely to contribute to the signalling from the inflamed gut to the brain. Cerebral adaptation processes induced by colitis are thought to be responsible for the disturbances in the molecular and behavioural manifestations of the stress response. Our findings provide new insights into the mechanisms that may underlie the association of IBD with psychiatric disorders and aberrant brain function.

## Additional Information

**How to cite this article**: Reichmann, F. *et al*. Dextran sulfate sodium-induced colitis alters stress-associated behaviour and neuropeptide gene expression in the amygdala-hippocampus network of mice. *Sci. Rep.* **5**, 9970; doi: 10.1038/srep09970 (2015).

## Figures and Tables

**Table 1:** Primers used for real-time reverse transcription PCR.

**Gene**	**Sequence forward primer (5’ → 3’)**	**Sequence reverse primer (5’ → 3’)**	**Product size (bp)**
NPY	CAGATACTACTCCGCTCTGCGACACTACA	TTCCTTCATTAAGAGGTCTGAAATCAGTGTC	106
NPY1R	TGATCTCCACCTGCGTCAAC	ATGGCTATGGTCTCGTAGTCAT	127
NPY2R	TCCGGGAATACTCCCTGATTG	GCAAAACGTACAGGATGAGCAG	135
CRH	GAATTTCTTGCAGCCGGAGC	CAGCGGGACTTCTGTTGAGA	104
BDNF	GTGACAGTATTAGCGAGTGG	TTCTCTAGGACTGTGACCGT	85
NR3C1	GACTCCAAAGAATCCTTAGCTCC	CTCCACCCCTCAGGGTTTTAT	109
PPIA	TTCCAGGATTCATGTGCCAG	CCATCCAGCCATTCAGTCTT	202

**Table 2:** Effects of colonic inflammation and acute psychological stress on plasma and brain cytokine levels.

	**Untreated**		**DSS**	
	**no WAS**	**WAS-90**	**no WAS**	**WAS-90**
Cytokines (Plasma)				
IL-1ß	n.d.	n.d.	n.d.	n.d.
IL-6	1.8±1.84	n.d.	**79.8±21.01 §§§**	**95.1±12.30 ###**
IL-17A	n.d.	n.d.	1.5±0.51	**1.6±0.42 ##**
IL-18	199.7±21.47	201.3±28.88	**1187.8±200.17 §§§**	**850.3±140.14 ###**
TNF-α	n.d.	n.d.	**4.1±1.25 §**	**4.7±1.21 ##**
GRO-α	25.6±1.96	25.1±4.30	**364.6±95.53 §§§**	**394.4±82.42 ###**
				
Cytokines (Brain)				
Hypothalamus				
IL-1ß	1.8±0.25	1.5±0.20	1.6±0.17	1.3±0.07
IL-6	0.9±0.13	1.1±0.15	1.6±0.17	**2.3±0.35 #**
IL-17A	0.8±0.20	0.9±0.18	1.0±0.13	0.9±0.13
IL-18	25.2±1.72	24.7±1.05	25.5±0.68	25.8±1.2
TNF-α	2.8±0.37	2.3±0.27	2.5±0.07	1.9±0.27
GRO-α	1.1±0.05	1.2±0.10	**1.6±0.20 §**	**2.4±0.28#**
				
Amygdala				
IL-1ß	1.4±0.37	1.7±0.29	1.9±0.13	2.5±0.40
IL-6	0.6±0.13	0.4±0.07	0.9±0.07	**1.3±0.23 #**
IL-17A	0.3±0.04	0.3±0.03	0.3±0.01	0.3±0.03
IL-18	21.1±6.21	17.8±2.65	14.2±0.87	15.0±0.64
TNF-α	0.2±0.13	0.3±0.17	0.2±0.13	0.6±0.17
GRO-α	0.6±0.05	0.6±0.07	0.8±0.13	1.4±0.24
				
Hippocampus				
IL-1ß	1.8±0.23	1.5±0.09	1.5±0.15	1.6±0.10
IL-6	0.5±0.03	0.5±0.02	0.9±0.12	**1.6±0.21 #**
IL-17A	0.6±0.05	0.6±0.01	0.5±0.04	0.6±0.03
IL-18	17.0±1.61	16.3±0.70	14.8±0.62	16.1±0.69
TNF-α	0.5±0.15	0.4±0.11	0.5±0.15	0.5±0.05
GRO-α	0.6±0.05	0.6±0.05	0.9±0.08	**1.5±0.36 #**

*Mice were treated for 7 days with dextran sulfate sodium (DSS; 2%, added to the drinking water) to induce colitis, while control animals received plain drinking water (Untreated). Parameters were measured in unstressed mice (no WAS) and 90 min after exposure to water avoidance stress (WAS-90) at the end of the 7-day treatment. The cytokine concentrations in plasma and brain are expressed as pg/ml and pg/mg, respectively. Data are presented as means*±*SEM, n = 10/group (plasma samples) and n = 5/group (brain samples). Significant differences are highlighted by bold letters:*^*§*^*P < 0.05,*^*§§§*^*P < 0.001 versus Untreated/CO;*^*#*^*P < 0.05,*^*##*^*P < 0.01,*^*###*^*P < 0.001 versus Untreated/Stress. n.d. = not detectable.*
